# Revising the Economic Imperative for US STEM Education

**DOI:** 10.1371/journal.pbio.1001760

**Published:** 2014-01-14

**Authors:** Brian M. Donovan, David Moreno Mateos, Jonathan F. Osborne, Daniel J. Bisaccio

**Affiliations:** 1 Stanford Graduate School of Education, Stanford University, Stanford, California, United States of America; 2 Centre D'Ecologie Fonctionnelle & Evolutive-CNRS (UMR 5175), Montpellier, France; 3 Department of Education, Brown University, Providence, Rhode Island, United States of America; University of California Berkeley, United States of America

## Abstract

An essential economic imperative for US STEM education is empowering students to reduce ecological degradation to improve economic welfare.

## Summary

Over the last decade macroeconomic studies have established a clear link between student achievement on science and math tests and per capita gross domestic product (GDP) growth, supporting the widely held belief that science, technology, engineering, and math (STEM) education are important factors in the production of economic prosperity. We critique studies that use science and math tests to predict GDP growth, arguing that estimates of the future economic value of STEM education involve substantial speculation because they ignore the impacts of economic growth on biodiversity and ecosystem functionality, which, in the long-term, limit the potential for future economic growth. Furthermore, we argue that such ecological impacts can be enabled by STEM education. Therefore, we contend that the real economic imperative for the STEM pipeline is not just raising standardized test scores, but also empowering students to assess, preserve, and restore ecosystems in order to reduce ecological degradation and increase economic welfare.

**Figure:**
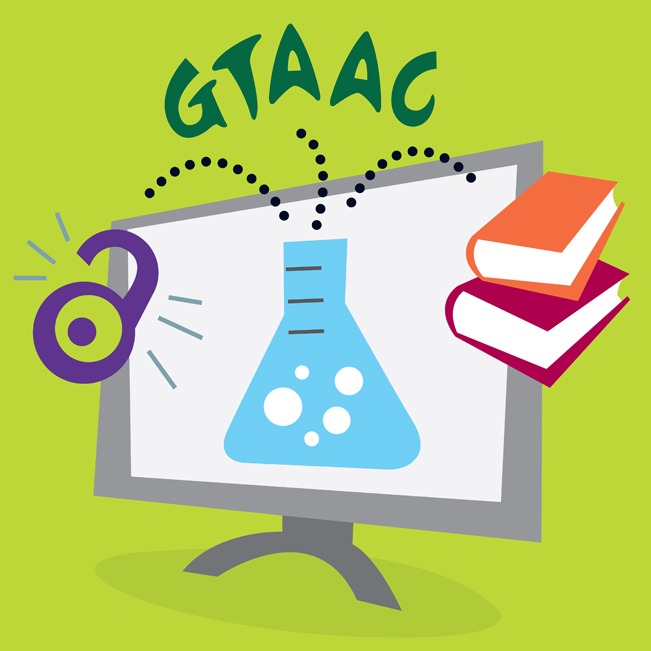
This Perspective is part of the Education Series

*This Perspective is part of the Education Series*

The economic imperative for STEM education is one of many different justifications for the teaching of science, but it is one of the most influential [1]. Advanced economies need to innovate, the argument goes, in order to grow their GDP and, therefore, need a continuous supply of scientists and engineers to drive innovation [1]–[3]. STEM education is the pipeline that provides these future scientists. Without this steady flow of scientists, policy makers and academics have argued US economic competitiveness will decline [4]. Consequently, raising student achievement on standardized science and math tests has become an economic imperative for education [2].

Our contention, however, is that the economic imperative for STEM education ignores not only the damage market economies often inflict on biodiversity and ecosystem functionality but also the negative consequences of these impacts on future economic welfare, including the costs of restoration. Moreover, economic activity is subsidized by multiple ecosystem services, such as crop pollination provided by insects or water purification provided by wetlands that are often overlooked in economic modeling. We argue that because economic modeling correlating STEM achievement tests to per capita GDP growth ignores the ecological consequences of economic growth, predictions of the future value of STEM education using achievement tests and GDP are flawed. And by teaching the knowledge and skills that allow drastic manipulations of the environment STEM education can indirectly enable ecological degradation. Given that well preserved ecosystems with higher biodiversity render more ecosystem services to society [5],[6], we contend that a major economic imperative for STEM education should be to empower students to assess, preserve, and restore ecosystems.

## Presumed Economic Benefits of STEM Education

Education contributes to economic growth by producing human capital. Student performance on standardized tests, such as the Programme for International Student Assessment (PISA) and Trends in International Mathematics and Science Study (TIMSS), has emerged as a measure of the cognitive skills and knowledge that constitute human capital [7]. Because they are highly correlated [7], TIMSS and PISA results are commonly aggregated by country into a single variable and used to predict national GDP growths. For example, Hanushek and colleagues [2] looked at the relationship between a country's standardized test scores and per capita GDP growth from 1960 to 2000 and found that countries with higher test scores in 1960 experienced greater GDP growth than countries with lower test scores in the subsequent decades after 1960. The authors argued that these correlational results demonstrate the value of science-related human capital for GDP growth [2],[7],[8] going so far as to contend that if the United States had raised its PISA test scores by 50 points during the 1990s, the American economy would have experienced sufficient economic growth to pay for the entire US education system in 2015 and thereafter. Indeed, it is a widely held belief that the cognitive skills measured by science and math achievement tests have some causal influence over GDP growth because scientists and engineers produce knowledge and innovations that lead to new markets, new jobs, and future consumption [3]. But assigning such extraordinary predictive value to science and math tests assumes a narrow view of economic growth. Moreover, such predictions are arguably based upon flawed assumptions about the relationships between STEM knowledge, the economy, and the environment.

## The Questionable Assumptions of the Economic Imperative

Traditionally, macro-economic models have not considered the ecological consequences of economic growth or their effects on future economic growth [9]. This relationship, modeled by The Environmental Kuznets Curve (EKC) [9], hypothesizes that as incomes rise, so does environmental degradation but that degradation declines with further income growth. Under this model society can outgrow environmental problems simply by raising average income.

The EKC, however, is not supported by what is known about many forms of ecological degradation [9],[10]. Data demonstrate that as incomes rise, most pollutants and flows of waste increase monotonically, thus signaling prolonged global environmental degradation [9],[10]. Further, the model assumes that any environmental harm from economic activity would not impede future economic growth [9]. Theoretical analyses show, however, that neither the marketplace nor technological innovation reverse ecological degradation because economic markets currently undervalue the natural capital embodied in ecosystems [10]. Indeed, raising income is unlikely to break destructive consumption patterns of natural resources because increased capital and increased exploitation of resources tends to go hand in hand, limiting future economic growth by damaging ecosystems [11],[12]. For example, as GDP increased between 1950 and 2005 in the United States, the amount of biologically productive land available for resources and waste absorption, or biocapacity, decreased [13].

Biocapacity decreases as a function of the degradation of the biodiversity and functionality of terrestrial, aquatic, and marine ecosystems. The organisms in ecosystems (for example, fish stocks) are a source of natural capital for humans, and the interactions between them, and other abiotic factors, provide ecosystem services to humans. However, the economic value of ecosystems is often established only after they have sustained damage [14],[15]. For example, American horseshoe crabs (*Limulus polyphemus*) are bled non-destructively to produce amoebocyte lysate, which is used to detect bacterial endotoxin that causes septic shock and death in humans [16]. Despite the clear benefit of this stock of capital to human welfare and the biomedical industry, horseshoe crab populations have been decimated over the last 20 years by overfishing and other human induced causes [16]. A broader estimate of the value of ecosystem services indicates that the entire biosphere provides on average US$58 trillion (estimated 2013 US$ value) of ecological subsidies per year [17]. New Jersey wetlands, for example, provide disturbance regulation at the rate of US$3 billion per year [18]. Likewise, by investing US$1.5 billion on watershed protection, New York City avoided US$6 billion in water treatment costs [19].

Wetland destruction also provides a unique perspective for examining the purported economic benefits of STEM education [i.e.], [2,8]. Approximately 5,119,000 hectares of wetlands were destroyed between the mid-1950s and the late 1990s in the US [20], and it is estimated that each hectare of wetland produces US$14,785 worth of ecosystem services each year [17]. Thus, the total cost of US economic development in the latter half of the twentieth century, in terms of wetland destruction, could be estimated as US$135 billion (estimated 2013 US$ value). Yet this cost of wetland damage does not figure into models of the future economic value of STEM education [i.e.], [2,8], even though these damages occurred during the period of economic development upon which the models of its value were based. Indeed, reviews of the modeling methods used in studies on the relationship between STEM based human capital and GDP growth include no discussion of how to correct per capita GDP estimates for the ecological costs of economic development [7], such as reductions in US biocapacity [13].

But, if STEM education produces STEM based human capital—capital that is responsible for technological innovations and economic growth—what responsibility does STEM education bear for the economic costs associated with reductions in US biocapacity? The same science related human capital that allows one to improve the productivity of farming, construct new dams, engineer urban sprawl, or produce new chemicals is the same human capital that reduces the biocapacity of wetlands [19]. And when markets undervalue natural capital, the same human capital that produces technological innovations can, and often does, result in the rapacious consumption of natural capital [10]. Furthermore, the standard environmental education model offers little promise of remediation because it often operates upon inaccurate models of human behavior change and so often fails to produce the lifestyle changes that reduce ecological degradation [21]. Thus, STEM education creates future economic costs by teaching the knowledge and skills that enable ecological degradation, albeit unintended, while failing to promote the kinds of behaviors that might effectively mitigate such degradation.

It is simply untenable to predict the future value of STEM education over the next 80 years [e.g.], [2,8] without considering the ecological degradation that can ensue from enhanced economic activity and its consequences for human welfare [9]–[13],[22]. Since history shows that ecological degradation is brought about by the economic activities that are a product of science and technology that are supported by STEM education, it is a paradox that ecological externalities are discussed pervasively in environmental economics yet so absent from discussion of the economic consequences of STEM education. Thus, we contend that an education that fails to acknowledge the ways in which STEM knowledge might impede economic growth indirectly by enabling ecosystem degradation is, at best, guilty of ignorance and, at worst, deception.

This is *not* to argue that we should not teach STEM subjects, or that STEM education has not increased public understanding of environmental problems, or that there is no good economic rationale for STEM education. For example, increasing the representation of women and people of color in the STEM pipeline to improve their access to better jobs is a worthy economic imperative for STEM education [3]. Likewise, the integration of environmental justice [23], climate change [24], and socio-scientific issues [25] into school science arguably improves environmental literacy. But all too often, economic rhetoric about the value of STEM education results in the imperative to raise achievement on science and math tests [i.e.], [2,8]. When this occurs, curriculum and instruction in school science can become myopically focused on the transmission of a body of disconnected and decontextualized facts [1]—facts that are only important because they need to be mastered in order to be successful on the next test within the STEM pipeline. It is this kind of education that does not afford students the opportunity to think deeply or critically about their life in relationship to STEM, the economy, and the environment.

Rhetoric about the economic value of STEM education is all the more concerning because GDP is a flawed indicator of economic welfare. As Kubriszewski and colleagues [13] point out, GDP was never designed to measure economic welfare. Other indicators, like the Genuine Progress Indicator (GPI) and the Index of Sustainable Economic Welfare (ISEW), measure economic welfare produced through economic activity. GPI, for example, adjusts the personal consumption component of GDP using measures of environmental degradation and income inequality to create a better approximation of the sustainability of economic growth [13]. By comparing GPI and GDP growth amongst 17 countries representing 53% of the world population between 1950 and 2005, Kubriszewski and colleagues [13] found that GDP growth improves human welfare to a threshold point of US$7,000 per capita GDP (reached in 1978), after which further increases in GDP growth are associated with decreased economic welfare (i.e., GPI), in part, because of impacts on ecosystems. Thus, we contend that the real economic imperative for US STEM education in the 21st century is not teaching to the test to increase GDP, rather it is teaching the knowledge and skills that might increase economic welfare.

## Producing Economic Welfare through STEM Education

One way an economically motivated STEM education policy can increase economic welfare is by reducing environmental degradation. Toward that end, STEM education must teach students about the benefits of biodiversity and ecosystem functions [14]–[19],[22] to redress the devaluation of ecosystems [10]–[15],[17]. Furthermore, it should teach students how to assess, preserve, and restore ecosystems in their local communities because research suggests that large increases in biodiversity and ecosystem services can result from restoration efforts carried out locally [26].

To promote a nuanced view of environmental issues, this curriculum should stress that economic growth can increase human welfare up to a point, after which human welfare can deteriorate along with ecosystems [13]. We are not suggesting that lessons pit economy against ecology, but that they challenge students to envision social and technological solutions to environmental problems that are economically feasible. By the same token, these solutions should not overlook the fact that the burden of environmental degradation falls disproportionately on the poor and people of color [11],[27],[28]. Thus, the curriculum should ask students to envision equitable solutions to environmental problems. Finally, research suggests that introducing students to careers in science [29],[30] and building science related social relationships enhances students' STEM career aspirations [29],[30]. Consequently, the STEM curriculum should explicitly introduce students to gainful “Green Jobs” [31] as well as individuals who have these jobs, in order to increase the likelihood that students pursue STEM career tracks that contribute to a sustainable economy.

Our revision of the economic goals of STEM education is therefore project-based and interdisciplinary (see Table 1). It requires the collaboration of researchers, science educators, and students on projects directed towards the assessment, preservation, and restoration of ecosystem services found within the geographical reach of a school community in order to improve human welfare. The knowledge of researchers will be needed to ensure the collection of quality data that can be used for the purposes of ecosystem service management [15]. The knowledge of educators will be needed to construct a valuable educational experience using this data—an experience that is different from lab work, which rarely requires students to integrate scientific concepts with phenomena using a scientific model [32], and an experience that would engage students in collaborative and critical discourse [33] to understand the scientific and economic complexities inherent in environmental problems. The knowledge that students possess about their home communities is needed to communicate effectively the findings of these projects to diverse audiences. Cooper and colleagues [34] outline community science research models with high research, management, and education value that could be used to organize these field projects. Likewise, Kloser and colleagues [35] propose a framework for integrating research into teaching that could be used to organize classroom instruction for these projects, which, if used, can also improve students' competency at experimental design and data interpretation [36].

**Table 1:** Suggested objectives and projects for STEM education at each grade level.

Grade Level	Objectives and Rationale	Examples of Interdisciplinary Projects
*Elementary school*	Build positive attitudes towards non-human organisms by stressing how they help humans because pro-environmental attitudes are an important baseline predictor of pro-environmental behavior [21].	(i) Learn about organisms essential to food production and carry out descriptive studies on them in school gardens. Then, communicate findings to the school community.(ii) Learn about the cultural practices that local indigenous peoples use(d) to manage natural capital and have older students educate younger students about how and why these groups value(d) non-human kinds.
*Middle school*	Teach in depth about one local ecosystem service and its benefits because particular attitudes towards specific environmental problems predict whether one engages in environmental behavior [21]. Introduce students to green jobs and local professionals who have them in order to lay the foundation for a sustainable economy.	(i) Monitoring of a local ecosystem service where students work collaboratively to choose appropriate sampling techniques, analyze and interpret data, argue about its meaning, and effectively communicate these interpretations to different audiences outside of school (i.e., local politicians, business people, family).(ii) An interview project where students are introduced to local professionals with green jobs and interview them to learn about the job. Students present their findings to the class.
*High school*	Teach particular strategies used to solve environmental problems and have students apply these strategies to a local environmental issue, because: (i) knowledge of environmental action strategies reinforces the relationship between pro-environmental attitudes and pro-environmental behavior [21]; (ii) building upon self-efficacy and locus of control can influence the desire to engage in newly developed environmental behaviors [21].	(i) Design and carry out adaptive co-management projects [34] around a local ecosystem service in conjunction with a local scientist or through a MOOC offered by a university.(ii) Discuss the sociological dimensions of ecological degradation, such as EJ issues [28]. Then, envision solutions to local EJ issues and present them to local policy makers in the community.(iii) Apply environmental action strategies to address a local environmental issue in collaboration with local STEM professionals who are environmental advocates.
*Undergraduate*	Deepen students' awareness of the complex relationships inherent in human-ecological interactions in order to create a new generation of STEM workers who are capable of working across disciplinary boundaries to assess, preserve, and restore ecosystems in order to improve human welfare.	(i) Participation in faculty led research programs that investigate the relationship between biodiversity and ecosystem services, restoration ecology, conservation biology, environmental economics, and social ecology.(ii) Engage mathematics students in projects that model the non-linear relationships between population growth, poverty, consumption, and ecology [10].(iii) Engage social science students in projects that require them to envision win-win solutions to biodiversity issues, which are interventions that can benefit both the rural poor and biodiversity [11],[27].(iv) MOOCs that engage participants in ecological research using community science research protocols [34] and which also require people to adopt behaviors to reduce their ecological footprint.

EJ, environmental justice.

Our proposals could be incorporated into existing educational initiatives. The Globe Program, for instance, brings scientists, educators, and students together on research projects that investigate the environment [37]. Similarly, the HabitatNet program engages high school students in biodiversity monitoring of tropical and temperate forests [38] through participatory action research [34]. Both of these programs train teachers to use established field research protocols with students. Such programs could be adapted to train teachers to empower students to assess, preserve, and restore ecosystems. Afterwards, long-term teacher support could be offered through Massive Online Open Courses (MOOCs). Furthermore, initiatives like the Ecology Society of America's SEEDs program, which seeks to increase the diversity of the ecology profession through ecology club opportunities that promote ecological awareness and action [39], might be used as a platform to organize the ecological research projects that are integral to our plan. For example, undergraduates and professors involved in SEEDs could work with school science programs to design adaptive co-management research projects [34] in local ecosystems. Finally, our revision of STEM is aligned with the Next Generation Science Standards [40], thus making it easy to assimilate within contemporary K–12 science instruction.

## Conclusion

Justifying STEM education through the economic imperative demands a consideration of what the limitations of this imperative might be. The purported relationship between STEM education and economic growth rests upon the questionable assumption that economic development has no ecological costs or that those costs can be eliminated through continued GDP growth. A good education enables students to live an economically, socially, culturally, and politically responsible life. It helps students to put their lives in order, which means knowing which things are more important, or as important, as other things. If science and technology facilitates ecological degradation, then an essential economic imperative for 21st century STEM education is making students aware of the possible outcomes of their actions as scientists and technologists and empowering students to assess, preserve, and restore ecosystems, and hence the services they render to society. To deny that human civilization is dependent upon nature, and thus, to dismiss the ecological costs of economic activity, can only further undermine our children's future economic welfare.
